# Food-grade titanium dioxide and zinc oxide nanoparticles induce toxicity and cardiac damage after oral exposure in rats

**DOI:** 10.1186/s12989-023-00553-7

**Published:** 2023-11-17

**Authors:** Manuel Alejandro Herrera-Rodríguez, María del Pilar Ramos-Godinez, Agustina Cano-Martínez, Francisco Correa Segura, Angélica Ruiz-Ramírez, Natalia Pavón, Elizabeth Lira-Silva, Rocío Bautista-Pérez, Rosina Sánchez Thomas, Norma Laura Delgado-Buenrostro, Yolanda Irasema Chirino, Rebeca López-Marure

**Affiliations:** 1 Departamento de Fisiología, Instituto Nacional de Cardiología Ignacio Chávez, Juan Badiano No. 1, Colonia Sección 16, Tlalpan, C.P. 14080 Ciudad de Mexico, México; 2 Departamento de Microscopía Electrónica, Instituto Nacional de Cancerología, Ciudad de México, México; 3 Departamento de Biomedicina Cardiovascular, Instituto Nacional de Cardiología Ignacio Chávez, Ciudad de México, México; 4 Departamento de Farmacología, Instituto Nacional de Cardiología Ignacio Chávez, Ciudad de México, México; 5 Departamento de Biología Molecular, Instituto Nacional de Cardiología Ignacio Chávez, Ciudad de México, México; 6 Departamento de Bioquímica, Instituto Nacional de Cardiología Ignacio Chávez, Ciudad de México, México; 7 Unidad de Biomedicina, Facultad de Estudios Superiores Iztacala, UNAM, Ciudad de México, México

**Keywords:** E171, Titanium dioxide nanoparticles, Zinc oxide nanoparticles, Apoptosis, Autophagy, Cardiac dysfunction

## Abstract

**Background:**

Metallic nanoparticles (NPs) are widely used as food additives for human consumption. NPs reach the bloodstream given their small size, getting in contact with all body organs and cells. NPs have adverse effects on the respiratory and intestinal tract; however, few studies have focused on the toxic consequences of orally ingested metallic NPs on the cardiovascular system. Here, the effects of two food-grade additives on the cardiovascular system were analyzed.

**Methods:**

Titanium dioxide labeled as E171 and zinc oxide (ZnO) NPs were orally administered to Wistar rats using an esophageal cannula at 10 mg/kg bw every other day for 90 days. We evaluated cardiac cell morphology and death, expression of apoptotic and autophagic proteins in cardiac mitochondria, mitochondrial dysfunction, and concentration of metals on cardiac tissue.

**Results:**

Heart histology showed important morphological changes such as presence of cellular infiltrates, collagen deposition and mitochondrial alterations in hearts from rats exposed to E171 and ZnO NPs. Intracellular Cyt-C levels dropped, while TUNEL positive cells increased. No significant changes in the expression of inflammatory cytokines were detected. Both NPs altered mitochondrial function indicating cardiac dysfunction, which was associated with an elevated concentration of calcium. ZnO NPs induced expression of caspases 3 and 9 and two autophagic proteins, LC3B and beclin-1, and had the strongest effect compared to E171.

**Conclusions:**

E171 and ZnO NPs induce adverse cardiovascular effects in rats after 90 days of exposure, thus food intake containing these additives, should be taken into consideration, since they translocate into the bloodstream and cause cardiovascular damage.

**Supplementary Information:**

The online version contains supplementary material available at 10.1186/s12989-023-00553-7.

## Background

Nanotechnology is used to manipulate matter in the manufacturing process of materials and products produced by nanotechnology are denominated nanomaterials. Nanomaterials contain nanoparticles (NPs) that have a size of < 100 nm and are employed for industrial purposes. NPs are currently used in the food industry to improve quality, shelf life, color, cost, safety, nutritional properties, and fitness. NPs are also used in food packaging, drug delivery systems, and antimicrobial treatment, among others [1]. The composition of NPs also includes inorganic materials such as metals, and NPs containing metals have many industrial applications. They are therefore present in a wide variety of commercial products. Metallic NPs containing silver, iron oxide, titanium dioxide, silicon dioxide, and zinc oxide are currently used in the food industry [2]. Titanium dioxide (TiO_2_) labeled as E171 is authorized in the United States of America (USA) as a food additive, and it is not considered as a nanoparticle because it only contains 40% of NPs (TiO_2_ NPs) and the rest is constituted by microsized particles of TiO_2_ [3, 4]. However, the potential immunotoxicity and capacity to cause inflammation, combined with the potential neurotoxicity renders this compound unsafe for health when used as a food additive [5]. The European Food Safety Authority (EFSA) published in May 2021 that E171 is not safe because can induce genotoxicity [5]. Despite this, it is still employed as a food additive in various countries of the world, since it provides a white color and opacity in foods like candies and chewing gum, coffee creamer, sauces, spreads, pastries, and icing. E171 is also used to give brightness to toothpastes, to enhance the flavor of other non-white foods and to clear beverages [6]. On the other hand, ZnO NPs are naturally white and thermostable materials used in beauty care, topical products, pigments, coatings, and electronic gadgets, among others [7]. ZnO NPs are currently used to improve food preservation and packaging properties given their antimicrobial properties [8].

E171 and ZnO are among the five most widely used nanomaterials, and human exposure to them has increased [9]. Major routes of exposure include inhalation, dermal contact in occupational settings and ingestion of foods containing them [10]. A decade ago, in USA and the United Kingdom (UK) it was revealed that children under 10 years old consumes 2–4 times more TiO_2_ NPs than adults (1–3 vs. 0.2–1 mg TiO_2_/kg bw/day, respectively) [6]. Human consumption of products containing NPs has dramatically increased actually and in a recent estimation, the daily maximum consumption of E171 by children is up to 32.4 mg/kg bw/day [11]. However, exposure to TiO_2_ depends largely on dietary habits, and in special cases it has been shown that the exposure is several hundreds of milligrams per day.

Entry by inhalation or ingestion is among the major concerns of oral exposure of NPs. This is followed by translocation into the systemic circulation affecting different organs. Another concern is the possibility of NPs crossing physiological barriers damaging the cardiovascular system [12]. Some studies on mice and rats showed that the orally administered ZnO NPs appeared in the blood and in several organs including liver, blood, brain, pancreas, kidneys and bones [13–16]. Another study revealed that TiO_2_ NPs are distributed into liver, spleen, kidneys, lungs, heart, brain, thymus, and reproductive organs after intravenous administration in rats [17]. In addition, another study showed that the oral administration for 30 and 90 days of TiO_2_ NPs dose (0, 2, 10, 50 mg/kg bw) can affect cardiac function and produce a strong inflammatory response [18]. ZnO NPs toxicity is based on its solubility leading to increased levels of zinc ions, while E171 toxicity is attributed to its accumulation after uptake since it is not degraded or eliminated by exocytosis [19].

Based on the above information, we hypothesized that the oral consumption of NPs could induce the damage of cardiac cells after chronic exposure. To test this, we assessed cardiac effects of orally administered E171 and ZnO NPs in male Wistar rats using an esophageal cannula for 3 months. Then, we assessed morphological changes in cardiac tissue, inflammation and cell death markers, expression of apoptotic and autophagic proteins, mitochondrial dysfunction, and concentration of metals in order to evaluate cardiac damage.

## Materials and methods

### E171 and ZnO NPs characterization

E171 was purchased from Mark Al Chemical de México (CAS number: 13463–67-7; color index 77,891) (Mexico City, MEX), and ZnO NPs (< 50 nm) were obtained from Sigma Aldrich (677,450) (St. Louis, MO, USA). The physicochemical characterization of E171 and ZnO NPs was performed in 3 different media; the first one was HEPES saline solution (150 mM NaCl, 10.9 mM HEPES, 4,4 mM KCl, 12.2 mM glucose, pH 7.4), the second was simulated gastric fluid, and the third one was simulated intestinal fluid. Simulated gastric fluid contained 80 mM HCl and 34 mM NaCl pH 1.1 [20]. On the other hand, simulated intestinal fluid contained 60 mM KH_2_PO_4_ and 20 mM NaOH pH 6.8 [21]. The primary shape of NPs, their hydrodynamic size, polydispersity index (PDI), and zeta potential were determined by dynamic light scattering with a Zetasizer nano‐zs90 equipment. Briefly, three independent experiments were performed and each experiment was done in triplicate. All data are presented as mean ± standard deviation. The equipment reads each sample and gives three lectures, one is the hydrodynamic size, the second is the zeta potential, and the third one is PDI. We run a separate measurement for E171 in HEPES saline solution, in simulated gastric fluid and in simulated intestinal fluid. Other similar measurements to ZnO NPs were performed. The values of pH of solutions used for physicochemical characterization are shown in Table 1.

**Table 1:** Physicochemical characteristics of E171 and ZnO NPs

Nanoparticle	Vehicle	pH	Hydrodynamic size (nm)	Zeta potential (mV)	Polydispersity index
E171	HEPES saline solution	7	1022 ± 186	− 13.18 ± 1.93	0.18 ± 0.15
	Simulated gastric fluid	1.1	677 ± 18.5	− 9.19 ± 3.05	0.57 ± 0.01
	Simulated intestinal fluid	6.76	1147 ± 70.8	− 12.16 ± 1.02	0.68 ± 0.21
ZnO NPs	HEPES saline solution	7	448.9 ± 42	− 4.3 ± 1.16	0.96 ± 0.1
	Simulated gastric fluid	1.1	571 ± 48	− 7.07 ± 0.07	0.65 ± 0.01
	Simulated intestinal fluid	6.76	479 ± 30.8	− 14.45 ± 3.1	1.0 ± 0.1

E171 and ZnO NPs (20 μg) were dispersed in 1 mL of HEPES saline solution, simulated gastric (pH 1.1) and intestinal (pH 6.76) fluid. All data are presented as mean ± standard deviation by triplicate of three independent experiments

Hydrodynamic diameter, zeta potential and polydispersity index were measured by the Zetasizer Nano-ZS90

The primary size of NPs was determined by transmission electron microscopy (TEM). For this, E171 and ZnO NPs were suspended in bi-distilled and filtered water. Subsequently, the suspension was emulsified and a 50 µL drop was placed on a copper grid with a formvar cover and 100 mesh carbon film for 10 min, then the grid was dried and observed with an electronic transmission microscope JEOL 10–10 equipped with a Hamamatsu camera system. Each type of particle was analyzed by duplicate.

### Animal protocol

The use of rats in the investigation protocol was approved by the Research Ethics Committee and Committee for Care and Use of Laboratory Animals of the Instituto Nacional de Cardiología Ignacio Chávez (protocol number 21–1274). All experiments were performed according to specifications of the Norma Oficial Mexicana NOM‐062‐ZOO‐1999.

Eighteen male rats (Wistar) were divided in three groups of 6 individuals and were administered HEPES saline solution (group I), E171 (group II), and ZnO NPs (group III). NPs were suspended in 1 mg/mL HEPES saline solution and kept at room temperature. Before use, NPs were vortexed at maximum speed for 5 min and then briefly between application in each rat.

It has been shown that the oral average consumption of E171 by a human is estimated to be around 5 mg/kg bw/day, and that this dose can reach different organs [17, 22, 23]. In this study rats received E171 at a dose of 10 mg/kg bw every other day for three months. Since ZnO NPs have showed a toxic effect around this dose in rats [24] and they are also a food additive, we consider using the same dose as E171. The two food additives were administrated orally using an esophageal cannula.

Rats had water and food *at libitum* and were weighed every week. At the end of the treatment, rats were anesthetized with 60 mg/kg bw pentobarbital administered intraperitoneally. An electrocardiographic register and the blood pressure measure were performed in all rats of each group; however, none significant difference was found between the different treatments compared with control rats (See Additional file 1: Fig. S1). A direct puncture to the left ventricle using a syringe of 10 mL was performed to obtain the total blood, which was centrifuged at 1200 rpm for 20 min to obtain the serum. After these procedures, the heart was removed and divided into three parts, one was intended for obtaining mitochondria, another for the determination of metals, and the last one for pathological and morphological analysis.

### Cell morphology

The tissue intended for histological staining was fixed in 4% paraformaldehyde in phosphate buffer solution (PBS) pH 7.4 for 1 h. Cross sections cuts (5 µm) were made in paraffin embedded heart tissue from the control and treated (E171 and ZnO NPs) groups. To analyze structural changes, hematoxylin–eosin (HE) and Masson's trichrome (TRCM) stains were performed. Numbers of infiltrated cells were quantified in HE slides. The integrated optical density (lum/pix^2^) of collagen deposits (blue) between myocardial fibers and perivascular region was measured in TRCM stains of heart cross-sections using the Image-Pro-9 software of Media Cybernetics. It was quantified in 4 fields (40X) of 4 cuts from different hearts for each group (*n* = 16).

Morphology was also analyzed by TEM [25]. To this, hearts were fixed in 2.5% glutaraldehyde-2.5% formaldehyde in PBS pH 7.4 for 1 h. Tissues were cut into 1 mm^3^ fragments and post-fixed with 2% OsO_4_, then dehydrated with alcohol and infiltrated with propylene oxide, and finally polymerized. Ultra-thin Sects. (70 nm) were cut and impregnated with uranyl acetate and lead citrate, then analyzed with an electronic transmission microscope.

### Inflammatory cytokines

Expression of inflammatory cytokines was measured in serum at the end of the study. After euthanasia, whole blood was collected, centrifuged for 15 min at 3500 rpm and serum was separated, frozen and kept at -70 °C until use. IL-6, IL-1β and TNF-α levels were measured by enzyme-linked immune sorbent assay (ELISA) kits (PeproTech) following manufacturer instructions. For this, 100 μL of each sample were added per well, the antibody for detection was diluted 1:5000 and proteins were revealed through streptavidin-peroxidase and O-phenylenediamine. Color development was measured at 490 nm, and a standard as a reference allowed for cytokine quantification.

### TUNEL assay

Cell death was measured by the TUNEL assay with the "In Situ Cell Death Detection Kit, Fluorescein", from Roche. Nuclei were stained with 4', 6-diamidino-2-phenylindole (DAPI). To calculate the percentage of apoptotic cells, total blue and green nuclei were quantified. Muscle fibers were labeled with wheat germ agglutinin texas red conjugated (WAG-TR-Molecular Probe).

### Mitochondrial proteins

At the end of the protocol, hearts of control, E171 and ZnO NPs groups were placed in cold isolation buffer containing 250 mM sucrose, 10 mM Tris–HCl, and 1 mM EDTA, pH 7.3. Whole hearts were homogenized and mitochondria were obtained by differential centrifugation, as previously described [26]. Mitochondrial total protein was quantified by the Lowry method [27].

### Apoptosis and autophagy markers

Expression of apoptosis- and autophagy-related proteins was detected by western blot in the mitochondrial protein obtained from the hearts derived from the different treatments. Cytochrome c (Cyt-C), Bcl-2, caspase-3 and caspase-9 were used as apoptotic markers, and Beclin-1 and LC3B as markers of autophagy. The following amount of mitochondrial protein was loaded in each line for different clots: 50 µg for Cyt-C, and 85 µg for Bcl-2, caspases, Beclin-1 and LC3B. Proteins were separated in 12% SDS–polyacrylamide gels (SDS-PAGE) and electro-transferred to Immun-Blot ®PVDF membrane (BIO-RAD). After transference, membranes were blocked with Tris-Buffer Saline (TBS) with 1% Casein (Bio-Rad) for 2 h at room temperature. Blocked membranes were incubated overnight at 4 °C in the same buffer containing rabbit monoclonal antibody against Cyt-C, Bcl-2, Beclin-1, and LC3B dilution (Abcam), and polyclonal antibodies against caspase-3 and caspase-9 (Abcam) all diluted 1:1000. Mouse monoclonal antibodies against Cox-IV and GAPDH (Abcam) were used at a dilution 1:1000 as loading controls. Membranes were rinsed with 150 mM NaCl, 25 mM Tris pH 7.4 + 0.1% Tween-20 (TBS-T) and then incubated with horseradish peroxidase coupled to an anti-rabbit or an anti-mouse IgG as secondary antibody (1:10,000). Bands were visualized using the enhanced chemiluminescence detection reagent (Clarity ™Western ECL, Bio-Rad) and exposed on Kodak Biomax ML scientific image film for 5 s. Band intensity was analyzed with the Gel Imaging System (UVP) and the Image J software. (Additional file 2: Fig. S2; Additional file 3: Fig. S2).

### Mitochondrial function

Mitochondrial oxygen consumption was measured using a Clark-type oxygen electrode (Yellow Springs Instruments, OH, USA). Experiments were carried out in 1.5 ml of basic medium containing 125 mM KCl, 3 mM Pi, pH 7.3. State 4 respiration was evaluated in the presence of 5 mM Glutamate plus 5 mM malate (substrates of complex I), recording started at 2 mg of mitochondrial protein. State 3 respiration was measured after addition of 200 µM adenosine diphosphate (ADP). Respiratory control index (RC) was calculated as the rate between state 3/state 4. The ADP/O Ratio, was calculated after addition of 200 µM ADP, measuring oxygen consumption during State 3.

### Quantification of metals

Quantification of 13 metals including Ti and Zn, was carried out in hearts from control and exposed rats. For this, 0.04–0.3 g (fresh weight) of heart were diluted in 1 mL ultrapure water and digested with 4 mL nitric acid at 90 °C in a dry bath during 3 h. Samples were analyzed by ICP-OES (inductively coupled plasma—optical emission spectrometry; Agilent, USA) using Argon as carrier gas [28].

### Statistical analysis

Differences between groups were evaluated by the Student's t test. Multiple comparisons were also performed by one-way analysis of variance (ANOVA) followed by Tukey´s pairwise comparison using the GraphPad Prism software version 5.01, and the differences of the mean of the groups were considered statistically significant when *p* < 0.05. Statistical analysis was performed with the software GraphPad Prism version 8.4.2.

## Results

### E171 and ZnO NPs characterization

NPs were suspended in saline solution and in two fluids to simulate the characteristic behaviors of E171 and ZnO NPs within the gastric and intestinal tracts. Table 1 summarizes physicochemical characteristics of E171 and ZnO NPs. E171 dispersed in simulated gastric fluid at pH 1.1 displayed aggregates of 677 nm ± 18.5 whereas in simulated intestinal fluid at pH 6.76 they formed larger aggregates of 1147 nm ± 70.8, while in HEPES saline solution they formed aggregates of 1022 nm ± 186. Zeta potentials of E171 in simulated gastric fluid, simulated intestinal fluid and HEPES saline solution were − 9.19 mV ± 3.05, 12.16 mV ± 1.02 and − 13.18 mV ± 1.93, respectively. On the other hand, ZnO NPs dispersed in simulated gastric fluid formed aggregates of 571 nm ± 48, in simulated intestinal fluid of 479 nm ± 30.8, while in HEPES saline solution of 448.9 nm ± 42.09. Zeta potentials of ZnO NPs in simulated gastric fluid, simulated intestinal fluid and in HEPES saline solution were − 7.07 mV ± 0.07, − 14.45 mV ± 3.10 and 4.3 mV ± 1.16, respectively (Table 1).

E171 suspended in HEPES saline solution, simulated gastric fluid or simulated intestinal fluid presented a PDI of 0.18 ± 0.15, 0.57 ± 0.01 and 0.68 ± 0.21, respectively; while ZnO NPs presented a PDI of 0.96 ± 0.1, 0.65 ± 0.01 and 1.0 ± 0.1, respectively (Table 1).

**Fig. 1:**
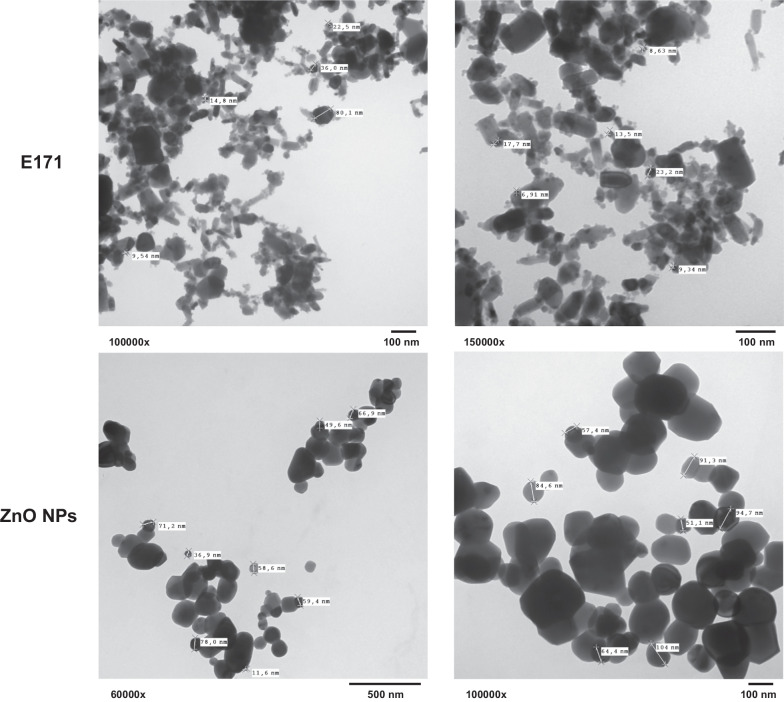
The primary size and shape of E171 and ZnO NPs obtained by TEM. E171 and ZnO NPs were suspended in bi-distilled water and 50 µL drop was analyzed in an electronic transmission microscope JEOL 10–10. Images are shown at direct magnification between 60,000 and 150000x

The primary size and shape of NPs was also determined by TEM. Results showed that E171 present a very heterogeneous mixture of particles of different forms and sizes ranging from 5 to around 120 nm, and tend to unite and form large aggregates. Results obtained from various spectra of E171, indicate that they are 100% composed by anatase [29]. On the other hand, the ZnO NPs had a more homogeneous shape and sizes ranging from around 30 to 250 nm (Fig. 1).

Previously, ZnO NPs were analyzed by X-ray diffraction by our work group and results showed that the crystalline phases correspond to zinc oxide according to Join Committee on Powder Diffraction Standards (JCPDS) [25].

### Nanoparticles increased infiltrated cells and collagen

**Fig. 2:**
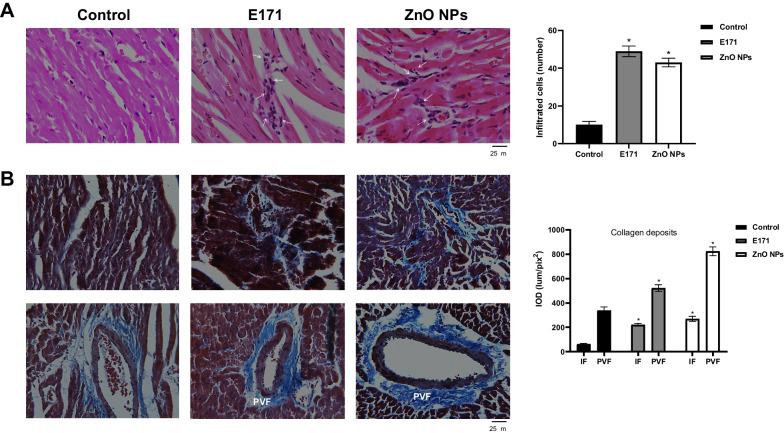
Nanoparticle exposure increased cell infiltrates and collagen deposits. Representative cross-sectional images of hearts derived from control rats, treated with E171 and ZnO NPs stained with HE **A** and Masson's Trichrome **B**. In A, arrows indicate areas with cell infiltrates. The graph shows average number of cells infiltrated in 4 fields of 4 sections of different rats from each group (*n* = 16). In B, interstitial fibrosis (IF) and perivascular fibrosis (PVF) are shown. Blue color indicates collagen deposits located between cardiac muscle fibers (IF) and perivascular region. The graph shows average integrated optical density (lum/pix^2^ × 10^4^) of the proportion of collagen deposits in 4 fields (40X) of 4 sections from different hearts of each group (*n* = 16). Mean ± SD of four rats is shown. * *p* < 0.05 versus control

During cell damage, inflammation is a predominant event. Endothelial cell integrity is lost and vascular permeability augments, facilitating leukocytes infiltration, accompanied by fibrosis in an inflammatory response leading to cardiovascular complications, including heart injury and vascular dysfunction [30]. Therefore, numbers of infiltrated cells and collagen deposits were counted. Hearts treated with both NPs exhibited a significant increase of cellular infiltrates (Fig. 2A). E171 and ZnO NPs augmented the number of infiltrated cells to 49 ± 3 and 43 ± 2, respectively, compared with control rats which had 10 ± 2. Both NPs also produced disorganized muscle fibers and broken fibers (Fig. 2B). Collagen fiber deposits (blue color) were located in these areas, indicating interstitial fibrosis (IF). To E171 the integrated optical density (IOD) was 220 ± 11 lum/pix^2^ and to ZnO NPs was 268 ± 20 lum/pix^2^ compared to control (67.4 5 lum/pix^2^). Blood vessels showed collagen deposits surrounding the outer region, such as perivascular fibrosis (PVF), with extensions between muscle fibers. To E171 the IOD was 523 ± 27 lum/pix^2^ and to ZnO NPs was 824 ± 36 lum/pix^2^ in comparison with control (338 ± 28 lum/pix^2^).

### Nanoparticle-induced cell morphological alterations

**Fig. 3:**
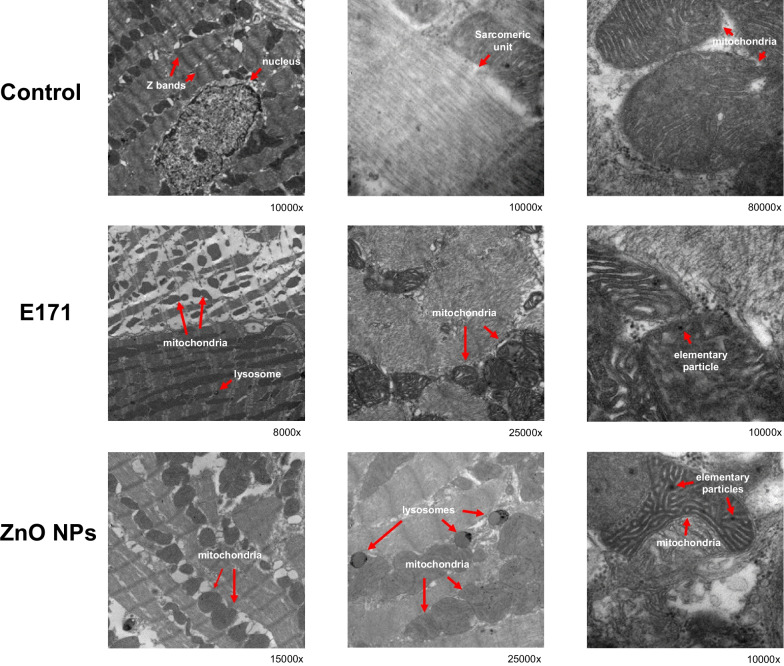
Nanoparticles altered cell morphology. Hearts were fixed, cut into sections and polymerized. Sections were analyzed with an electronic transmission microscope JEOL 1010 equipped with a Hamamatsu camera system. Images are shown at direct magnification between 8000 and 80000x. In control hearts, the integrity of the tissue (Z bands, nucleus, sarcomeric unit and mitochondria) is observed. E171 induced alteration mainly in mitochondria, while ZnO NPs induced the appearance of lysosomes and altered mitochondria). In hearts treated with NPs, the presence of elementary particles was observed

To obtain a detailed morphological structure, heart sections were analyzed by transmission electron microscopy. Control rat hearts showed mitochondria aligned in sarcomeric units, with well-defined Z lines, actin and myosin bands, with well-defined nucleus and nucleolus (Fig. 3). Mitochondria appeared well preserved with few elementary particles. Rats exposed to E171 displayed large areas with sarcomere disarrangement, loss of ultrastructural alignment, small disorganized mitochondria, elementary particles inside and some lysosomes. Rats exposed to ZnO NPs showed large spaces between sarcomeres with more mitochondrial aggregates and altered mitochondrial cristae. In addition, more elementary particles and lysosomes were detected.

### Nanoparticles had no effect on inflammatory cytokines release

**Fig. 4:**
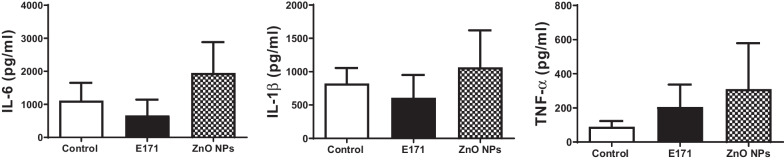
Nanoparticles did not induce the secretion of inflammatory proteins. Serum levels of IL-6, IL-1β and TNF-α (pg/mL) were quantified by ELISA at the end of the study. Mean ± SD of four rats is shown

More cellular infiltrates and collagen deposits were observed in hearts of rats exposed to both NPs, indicating inflammation. Overall tissue damage elevates cytokine production, pleiotropic molecules playing key roles in inflammatory responses involved in the pathogenesis of acute coronary syndromes and chronic heart failure, and both these responses are associated with cardiomyocyte loss [31]. Upon cardiac damage, serum concentrations of pro-inflammatory cytokines increase, including TNF-α, IL-6 and IL-1β. IL-1 can induce TNF-α synthesis in different cell types including myocytes, therefore these molecules were evaluated. Results showed no statistically significant changes in the expression of cytokines in rats exposed to E171 and ZnO NPs (Fig. 4); however, E171 and ZnO NPs showed a trend to increased TNF-α expression. There was a trend to higher levels of all cytokines in ZnO NPs exposure, suggesting a pro-inflammatory state eventually leading to cardiomyocyte loss or heart mechanical dysfunction.

### Nanoparticles induced cardiac cell death

**Fig. 5:**
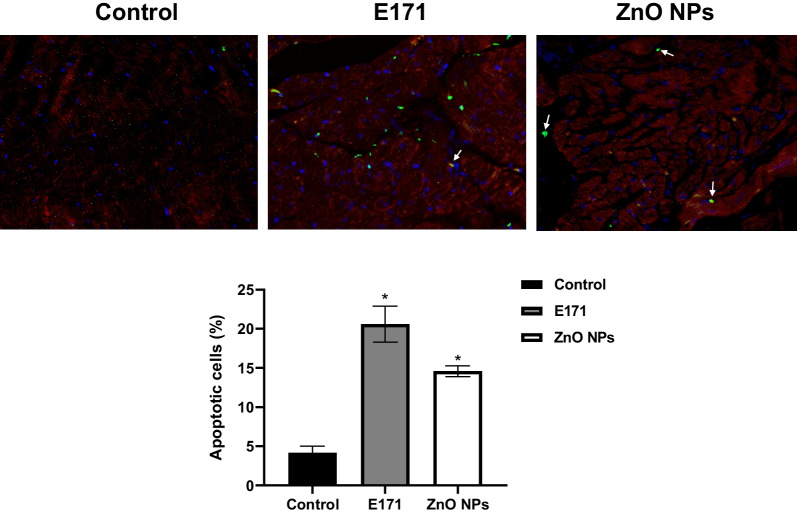
Nanoparticles induced apoptosis. Representative images of heart cross-sections of control rats, treated with E171 and ZnO NPs. Apoptotic rate was measured with the TUNEL assay (Green). Muscle fibers were delimited with wheat agglutinin texas red conjugated (WAG-TR) and nuclei were stained with 4', 6-diamidino-2-phenylindole (DAPI) (blue). The graph shows mean ± SD of the percentage of TUNEL-positive cells in 4 fields (40X) of 4 sections from different hearts of each group (*n* = 16). * *p* < 0.05 versus control

To evaluate whether nanoparticles promote cardiac cell death, we measured the number of TUNEL-positive cells. These were detected in hearts treated with both NPs, indicating apoptosis. An increase of 20% and 14% of apoptotic cells were obtained with E171 and ZnO NPs, respectively, in comparison with 4% of control (Fig. 5). Most TUNEL-positive cells were located in muscle fibers and some in circulating cells (arrows).

### ZnO NPs altered expression of apoptotic and autophagic proteins

**Fig. 6:**
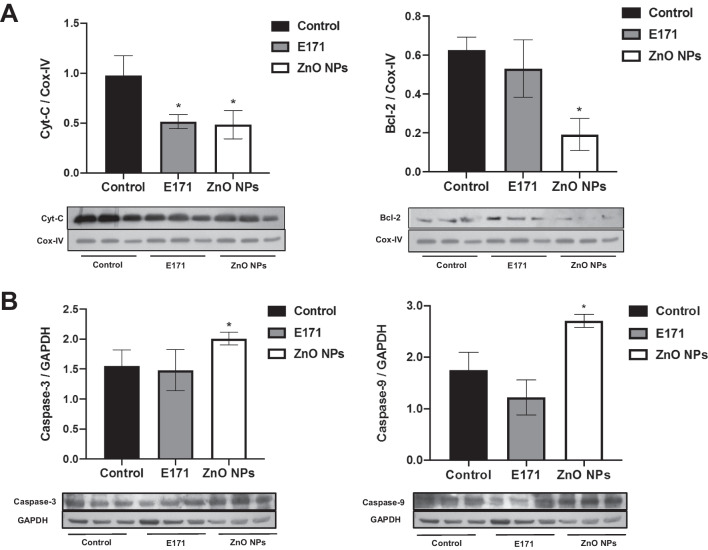
Nanoparticles altered expression of apoptotic proteins in mitochondria. Cyt-C and Bcl-2 expression **A** and caspase-3 and caspase-9 expression **B** of three independent rats of each group is shown. Cox-IV and GAPDH were used as loading control. A representative experiment of three performed of independent way is shown. The densitometric analysis shows the mean ± SD of three rats. * *p* < 0.05 versus control

**Fig. 7:**
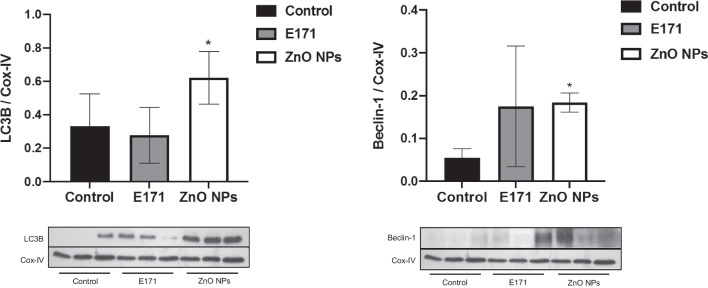
Nanoparticles altered expression of autophagic proteins in mitochondria. LC3B and Beclin-1 expression is shown. Cox-IV was used as loading control. Mean ± SD of three rats is shown. * *p* < 0.05 versus control

Since morphological alterations indicating damage and TUNEL-positive cells were observed, apoptotic and autophagic markers were evaluated in heart mitochondria. Treatment with E171 and ZnO NPs decreased by 50% Cyt-C content, a pro-apoptotic protein with respect to non-exposed rats (Fig. 6A). ZnO NPs significantly decreased by 70% Bcl-2 content, while there was increased caspase-3 and caspase-9 expression by 30 and 62%, respectively (Fig. 6B). ZnO NPs also significantly augmented by 100% and 200% LC3B and Beclin-1, respectively, in comparison with control rats (Fig. 7). In contrast, E171 did not affect Bcl-2 or caspase expression, and the same was found for autophagic proteins.

### Mitochondrial function

**Fig. 8:**
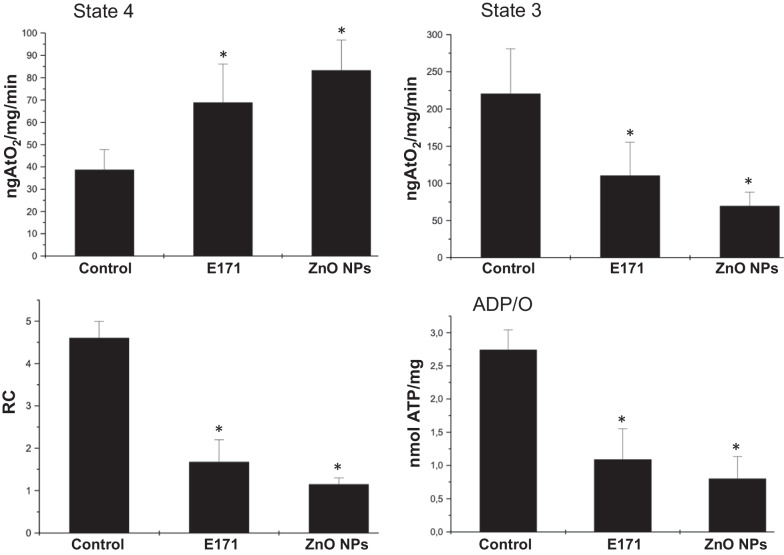
Nanoparticles altered cardiac mitochondrial function. Oxygen consumption was used to evaluate heart function. States 3 and 4 of respiration were measured. Respiratory control index (RC) was calculated as the ratio between state 3 and state 4 rates. The ADP/O ratio, was calculated after addition of 200 µM ADP measuring the amount of oxygen consumed during State 3. Mean ± SD of four rats is shown. * *p* < 0.05 versus control

In order to evaluate whether cellular alterations induced by NPs could alter cardiac function, we evaluated oxidative phosphorylation through basal oxygen consumption of cardiac mitochondria under the different treatments (Fig. 8). Oxygen consumption was accelerated by E171 and ZnO NPs, uncoupling mitochondria. This was verified since adding ADP as substrate for ATP synthesis increased oxygen consumption in untreated cardiac mitochondria but not in those exposed to nanoparticles. This basal oxygen consumption negatively affected respiratory control rate (RC) (i.e., mitochondrial coupling) and ATP synthesis.

### Nanoparticles increased calcium levels

**Fig. 9:**
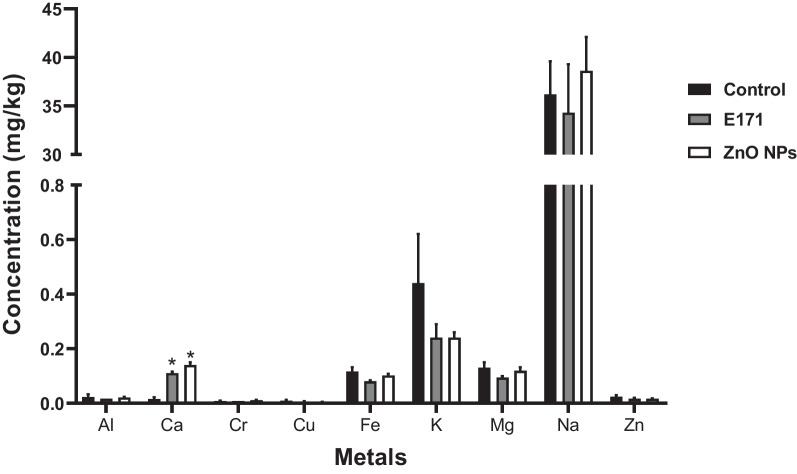
Nanoparticles increased calcium concentration. Concentrations of 13 metals were measured in cardiac tissue from either control or rats exposed to E171 and ZnO NPs. Tissues were analyzed by inductively coupled plasma optical emission spectrometry (ICP-OES). Manganese, selenium, molybdenum and titanium were not detected in hearts. Results are expressed as mean ± SD of three rats is shown. * *p* < 0.05 versus control

Since NPs can accumulate in different tissues and organs [32], we evaluated cardiac levels of titanium, zinc and other metals. NPs had no effect on concentrations of these metals except calcium that was significantly increased in rat hearts exposed to E171 and ZnO NPs (Fig. 9). Manganese, selenium, molybdenum and titanium were not detected in cardiac tissue.

## Discussion

Metal nanoparticles are widely used in the food industry. The most common are E171 and ZnO NPs, composed of inorganic materials such as titanium dioxide and zinc oxide, respectively. E171 contains around 40% of a nanoscale particle fraction (TiO_2_ NPs) and it is principally used as a whitening agent in candies, chewing gums, bakery goods and milk powders [1]. The size distribution of E171 used in this work is 50–100 nm: 38%; 100–150 nm: 34%; 150–200 nm: 16%, 200–250 nm: 12%. In other words, E171 has a 38% of NPs and 62% of MPs displaying an amorphous morphology [33].

ZnO NPs are zinc sources in supplements and functional foods, and they act as antimicrobial agents in food packaging [34, 35]. These NPs enter the body orally through food and translocate to the systemic circulation given their small size in the nanometric scale (< 100 nm), affecting different organs. They cause adverse effects and toxicity in different organs and cells, damaging DNA, causing inflammation and oxidative stress and inhibiting cell proliferation, among others [36]. We previously showed that ZnO NPs and E171 cause toxicity in rat cardiomyoblasts H9c2 [25]; therefore, understanding their toxicity in the cardiovascular system is fundamental. In this work, rats were orally exposed to E171 and ZnO NPs for three months and then, morphology, cell death and mitochondrial alterations of cardiac cells were evaluated.

It has been shown particle size and its surface area, play a key role in the biological effects of NPs; smaller size may exhibit higher biological interactions and adverse effects [37]. In our study, we measured hydrodynamic sizes of both NPs, and E171 showed twice larger aggregates when compared with ZnO NPs in HEPES saline solution (Table 1). TEM results showed a larger particle size of ZnO NPs compared to E171 (Fig. 1), and this size does not match the one indicated by the manufacturer (50 nm). We consider that the particle size of both NPs studied did not determine induction of toxicity in hearts from exposed rats, because ZnO NPs with the largest size were more harmful; however, the size of aggregate could be more relevant in the biological effect since ZnO NPs with a smaller aggregate size induced a higher harmful effect such as the induction of collagen deposits, although E171 which forms large aggregates and is composed by a mixture of nanoparticles and microparticles was also harmful. A previous study showed that both microparticles and nanoparticles of E171 have genotoxic potential [33].

The heterogeneity based on size of both types of NPs was assessed using the PDI. Polydispersity can occur due to size distribution in a sample or agglomeration or aggregation of the sample during isolation or analysis. Polydispersity index values rank from 0 to 1 and low values around 0.2 are related to uniform dissolution, while higher values reveal heterogeneity of size distribution [38]. NPs suspended in HEPES saline solution and both fluids had values ˃ 0.2 of PDI, indicating that the suspensions are not stable, containing several different size distributions of aggregates and settling particles, which coincide with the images obtained by TEM. NPs displayed the lowest value of PDI in simulated gastric fluid, and in simulated intestinal fluid displayed values closer to 1.0. Here, the influence on the solubility of ZnO NPs keeps a tight relationship with the pH of the solution, showing that a neutral pH does not favor the dispersion of particles as the acidic pH does. When E171 were suspended in low pH as in gastric fluids, their size changed, indicating size is influenced by pH and can impact cell internalization. Therefore, after oral consumption size variations ought to happen given pH variations along the different gut compartments. We found differences in PDI also influenced by pH. Zeta potential, which indicates the electrostatic charge and thus the charge repulsion/attraction between particles, can be affected by particle size and nature of solution [39]. High differences in zeta potential were observed in E171 and ZnO NPs depending on the vehicle and pH of solutions used, indicating changes in the stability of NPs, that can occur when NPs have contact with the gastric and intestinal fluids after their oral consumption, and that could influence their toxicity.

Histopathological examination of HE-stained cardiac tissues from rats exposed to E171 and ZnO NPs, showed infiltration of inflammatory cells such as leukocytes, and disorganized and broken muscle fibers (Fig. 2), indicating damage and local inflammation, which was not reflected in a significant increase in inflammatory cytokines in serum (Fig. 4). Cellular infiltrates have been observed for titanium NPs, in other models and organs. A study performed with TiO_2_ NPs found a leukocyte influx after 3–9 h of treatment using the air pouch model in CD1 mice and an increased production of chemokines [40]. In mice exposed to TiO_2_ NPs by intraperitoneal injection, many neutrophilic cells were found in the lung tissue [41]. Regarding ZnO NPs, higher number of inflammatory cells such as macrophages, lymphocytes and eosinophils were detected in mice lungs after pharyngeal aspiration [42]. Another study showed lobular and portal triads of infiltration with inflammatory cells in livers in rats exposed to ZnO NPs at a daily dose of 2 mg/kg bw for 21 days, indicating inflammation and oxidative stress [43]. Together these results suggest NPs are able to induce a strong inflammatory response in the exposed rats.

On the other hand, our results obtained with TRCM stain showed that cardiac tissue from rats exposed to both NPs presented more collagen deposits compared with control rats, indicating a fibrotic process resulting from previous chronic inflammatory reactions. Cardiovascular fibrosis can lead to organ failure and death [44]. Since evidence shows fibrosis and inflammation have different underlying mechanisms, it would be very interesting to determine which elements regulate these events and stimulate deposition of connective tissue destroying normal cardiac tissue architecture. Fibrosis induced by NPs has been observed mainly in the lung and liver. There are several studies reporting the effects of TiO_2_ NPs on liver where hepatic fibrosis was found. In some of them, mice were fed with 2.5, 5, and 10 mg/kg bw of TiO_2_ NPs for nine consecutive months and hepatic inflammatory cell infiltration and hepatic fibrosis were observed. These effects were mediated by the TGF-β/Smads/MAPK/Wnt signaling pathway [45]. This pathway was also involved in pneumonia and pulmonary fibrosis induced by TiO_2_ NPs in mice [46]. In other studies, TiO_2_ NPs induced alterations in the structure of the liver, infiltration of inflammatory cells, increase of collagen density and fibrosis in rats [47]. There is little information about the effects of ZnO NPs on fibrosis. Jacobsen and collaborators showed mice exposed once to NM-111 ZnO NPs by oro-pharyngeal aspiration had increased pulmonary collagen accumulation (fibrosis) [48]. To our knowledge, this is the first evidence of cardiac fibrosis induced by E171 and ZnO NPs.

The infiltration of inflammatory cells and fibrosis observed in hearts from ZnO NPs exposed rats was related to a tendency towards higher expression of all pro-inflammatory cytokines evaluated and although it was not statistically significant, it suggests the presence of an inflammatory response (Fig. 4). A substantial increase of IL-8 and stronger oxidant generation was observed in A549 human lung epithelial cells after treatment for 24 h with TiO_2_ NPs [49]. In these same cells, both TiO_2_ NPs and ZnO NPs produced a sustained inflammatory response (TNF-α, IL-10, and IL-6 release) and generation of ROS [50]. The capacity of ZnO NPs to increase inflammatory cytokines and ROS has been described both in vitro (THP-1 cells, human blood cells, macrophages) [51, 52] and in vivo (mice and rats) [53, 54], supporting the fact that NPs can trigger inflammatory responses. Elevation of these cytokines is linked to arrythmias or hyper-sensibility to calcium, an ion found at had high levels in our rats exposed to E171 and ZnO NPs (Fig. 9). Increases in calcium lead to malfunction and heart damage [44, 55]. Therefore, ZnO NPs could induce a serious cardiac damage. A study in rats exposed to TiO_2_ NPs for a long term (nine consecutive months) showed important changes in hearts such as titanium accumulation, infiltration of inflammatory cells and apoptosis of cardiac cells, reductions in net increases of body weight, and increase in heart indices of function [56]. They also found reduced ATP production in cardiac tissue, higher expression of NF-κB, IL-1β and TNF-α, and lower expression of anti-inflammatory cytokines. Therefore, TiO_2_ NPs might modulate cardiac functions [56].

The effects produced by E171 and ZnO NPs in hearts of exposed rats were strongly linked to cell death. Both NPs increased the number of Tunel-positive cells, indicating apoptotic cell death (Fig. 5). They also decreased drastically by 50% the levels of Cyt-C compared with mitochondria from control rats. In healthy cells, Cyt-C is located in the mitochondrial intermembrane/intercristae spaces [57]; therefore, these results suggest the permeabilization of the outer membrane induced by NPs promoting the mobilization and release of Cyt-C, to induces the proteolytic maturation of caspase-9 and caspase-3, which lead to cell death. Only ZnO NPs increased significantly caspases expression (Fig. 6), as well as levels of autophagic proteins (Fig. 7), showing activation of apoptosis and autophagy. It has been described that apoptosis and autophagy may be triggered by common upstream signals, and sometimes this results in combined autophagy and apoptosis [58]. Since a difference in the expression of evaluated proteins in mitochondria from rats exposed to the two NPs was observed, we hypothesized that E171 and ZnO NPs have different signaling mechanisms, probably because E171 is internalized, while ZnO NPs dissociate in Zn^2+^.

Mitochondrial dysfunction can be caused by mitochondrial reactive oxygen species (ROS) generation. The literature reports that oxidative stress plays a key role in NPs-induced toxicity [59]. Since ZnO NPs toxicity can be associated with formation of ROS and release of metal-ion, mitochondrial function was determined in the mitochondria derived from hearts after NPs exposure (Fig. 8). Preservation of mitochondrial function is essential for the supply of ATP to the organelles and metabolic pathways that depend on this source of energy [60]. Imbalances between oxygen consumption and ATP synthesis compromise energy supply and membrane permeability, releasing mitochondrial proteins into the cytosol (for example, Cyt-C), and activating pathways of programed death such as apoptosis. In our experimental conditions, both E171 and ZnO NPs modified bioenergetics, causing mitochondrial dysfunction. Failure derived from NPs could be causing Cyt-C release and triggering apoptosis (Figs. 5 and 6). Dysfunction mitochondrial is caused by uncoupling of the electron transport chain which results in enhanced production of ROS, depletion of cell ATP pool, extensive cell damage, and apoptosis of cardiomyocytes, causing cardiac dysfunction [61]. Mitochondrial malfunction obtained of the results of mitochondrial bioenergetics could be associated with the structural changes observed in the histological sections.

Based on the data obtained in this study, ZnO NPs were more toxic at the same concentrations when compared with E171. Similar results were obtained by Lai and collaborators, who tested toxicity of different metallic NPs on human neural cells and fibroblasts, where ZnO NPs were more effective than TiO_2_ NPs in inducing cell death [7]. A study performed on 19 different metal oxide NPs suggests inherent toxicity of released metal-ions is the key factor underlying their toxicity [58]. Upon entering the body, ZnO NPs are uptaken by cells and internalized either as free Zn^2+^ ions or as whole NPs [62]. Released Zn^2+^ ions cross the cell membrane by passive dissemination depending on the medium physicochemical properties (pH, UV illumination, exposure time, different components), and physiological properties of the ion (size, concentration, porosity, morphology), causing toxicity [63, 64]. Our TEM results did not show internalized whole ZnO NPs inside cardiac cells (Fig. 3); therefore, we suggest only Zn^2+^ ions are internalized. In a previous work by our group work, TEM showed that whole ZnO NPs are not internalized into rat H9c2 cardiomyoblasts; however, important morphological changes and dose-dependent toxicity are observed [25]. This suggests that the Zn^2+^ ion form is released causing the damage. This may be also due to their capacity to dissociate into Zn^2+^ ions A study with primary and immortalized immune cells exposed to ZnO NPs, indicated cell death caused by free Zn^2+^ ions is abrogated upon reduction by EDTA or CaCl_2_ [65]. All these results support that the Zn^2+^ ion form could be responsible for the effects of ZnO NPs observed in our exposed rats.

Finally, our results did not show the presence of Zn and Ti metals in hearts from exposed rats (Fig. 9); however, an increase in the levels calcium was observed, which was related to mitochondrial damage. This suggests that the toxic effect induced by both NPs is not direct but that it occurs through an indirect way. Different organs and cells can be activated and are responsible of the release of oxidant and inflammatory molecules and of other protein stress mediators, that may translocate the barriers and disrupt the tissue homeostasis in places apart from the exposure/accumulation sites [66]. A study showed that mediators secreted from activated microglial cells can damage neuronal cells, and tissue far away activating microglial cells and leading to a positive activation loop [67]. A similar behavior could be occurring in our in vivo model, where a large number of cells may be activated by the NPs and trigger signaling pathways of damage in cardiac cells.

Recently, the EFSA Panel on Food Additives and Flavourings (FAF) concluded that titanium dioxide can no longer be considered safe as a food additive due to its genotoxicity. They report that in spite of the low absorption of titanium dioxide particles after oral ingestion, they can accumulate in the body causing severe damage [5]. This observation coincides with the results obtained in this investigation.

In conclusion, our results reveal that chronic exposure to E171 and ZnO NPs in rats for at least three months changes cardiac tissue morphology, inducing fibrosis, cell infiltrate, cardiac cell death, and mitochondrial dysfunction. Therefore, the consumption of products containing these NPs would have to be considered, since it could be detrimental to human health and trigger the development of cardiovascular diseases.

### Supplementary Information

**Additional file 1 Fig. S1. **Electrocardiographic register **A** and blood pressure measure **B**. These measurements were performed by means of surgical procedure. To this, rats were anesthetized with sodium pentobarbital (60 mg/kg bw) and artificially ventilated through a cannula inserted into the trachea. One pressure transducer introduced in the femoral artery and three surface electrodes in DII, both connected to a SIEVART program led us to obtain the data of cardiac frequency (bpm) and blood pressure (mmHg) during a period of 10 minutes.

**Additional file 2 Fig. S2 **Original blots without cuts and with the molecular weight corresponding to 15, 25, 37, 50 and 75 KDa. Description of data: The antibodies used are specific for Cyt-C (14 KDa), BcL-2 (26 KDa), Caspase-3 (32 KDa) and Caspase-9 (46 KDa). In each lane, the mitochondrial sample of the 3 different conditions was loaded with 3 independent experiments.

**Additional file 3 Fig. S3.** Original blots without cuts and with the molecular weight corresponding to 15, 25, 37, 50 and 75 KDa. Description of data: The antibodies used are specific for LC3B (15 KDa) and Beclin-1 (52 KDa). The Cox IV (15 KDa) and GAPDH (36 KDa) was used as a loading control. In each lane, the mitochondrial sample of the 3 different conditions was loaded with 3 independent experiments.

## Data Availability

The datasets supporting the conclusions of this article are included within the article and its additional files.

## Abbreviations

ZnO NPs: Zinc oxide nanoparticles
E171: Food-grade titanium dioxide
HE: Hematoxylin–eosin staining
TRCM: Masson's Trichrome staining
TEM: Transmission electron microscopy
