# Supplementary material for: Food-grade titanium dioxide and zinc oxide nanoparticles induce toxicity and cardiac damage after oral exposure in rats
Source: Part Fibre Toxicol. 2023 Nov 17;20:43. doi: 10.1186/s12989-023-00553-7 (PMC10655394; doi:10.1186/s12989-023-00553-7)
Supplement: Supplementary file 1 — Additional file 1 Fig. S1. Electrocardiographic register A and blood pressure measure B. These measurements were performed by means of surgical procedure. To this, rats were anesthetized with sodium pentobarbital (60 mg/kg bw) and artificially ventilated through a cannula inserted into the trachea. One pressure transducer introduced in the femoral artery and three surface electrodes in DII, both connected to a SIEVART program led us to obtain the data of cardiac frequency (bpm) and blood pressure (mmHg) during a period of 10 minutes. [file 12989_2023_553_MOESM1_ESM.pptx]

## Slide 1
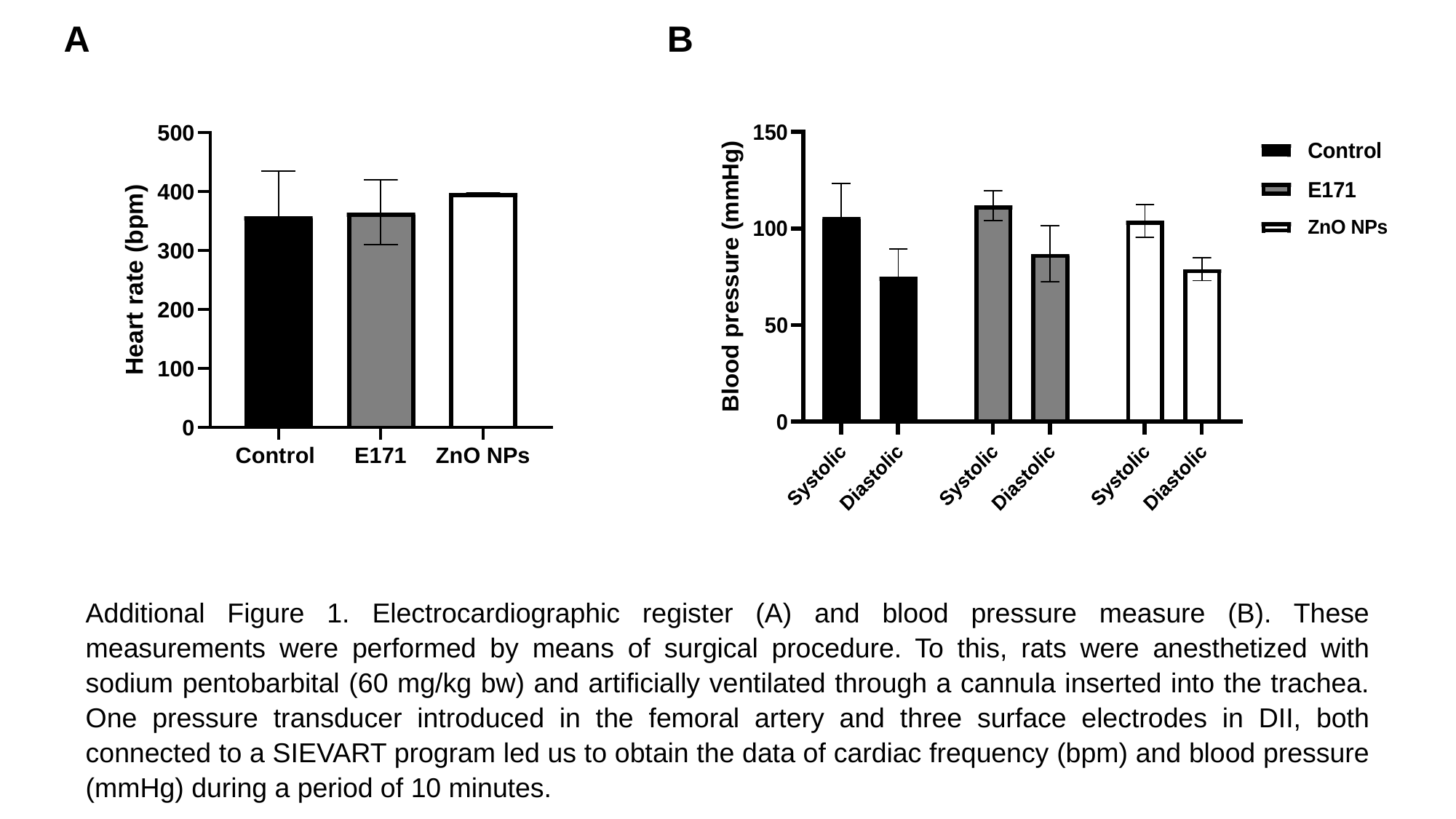

A B
Additional Figure 1. Electrocardiographic register (A) and blood pressure measure (B). These measurements were performed by means of surgical procedure. To this, rats were anesthetized with sodium pentobarbital (60 mg/kg bw) and artificially ventilated through a cannula inserted into the trachea. One pressure transducer introduced in the femoral artery and three surface electrodes in DII, both connected to a SIEVART program led us to obtain the data of cardiac frequency (bpm) and blood pressure (mmHg) during a period of 10 minutes.
