# Supplementary material for: Food-grade titanium dioxide and zinc oxide nanoparticles induce toxicity and cardiac damage after oral exposure in rats
Source: Part Fibre Toxicol. 2023 Nov 17;20:43. doi: 10.1186/s12989-023-00553-7 (PMC10655394; doi:10.1186/s12989-023-00553-7)
Supplement: Supplementary file 2 — Additional file 2 Fig. S2 Original blots without cuts and with the molecular weight corresponding to 15, 25, 37, 50 and 75 KDa. Description of data: The antibodies used are specific for Cyt-C (14 KDa), BcL-2 (26 KDa), Caspase-3 (32 KDa) and Caspase-9 (46 KDa). In each lane, the mitochondrial sample of the 3 different conditions was loaded with 3 independent experiments. [file 12989_2023_553_MOESM2_ESM.pptx]

## Slide 1
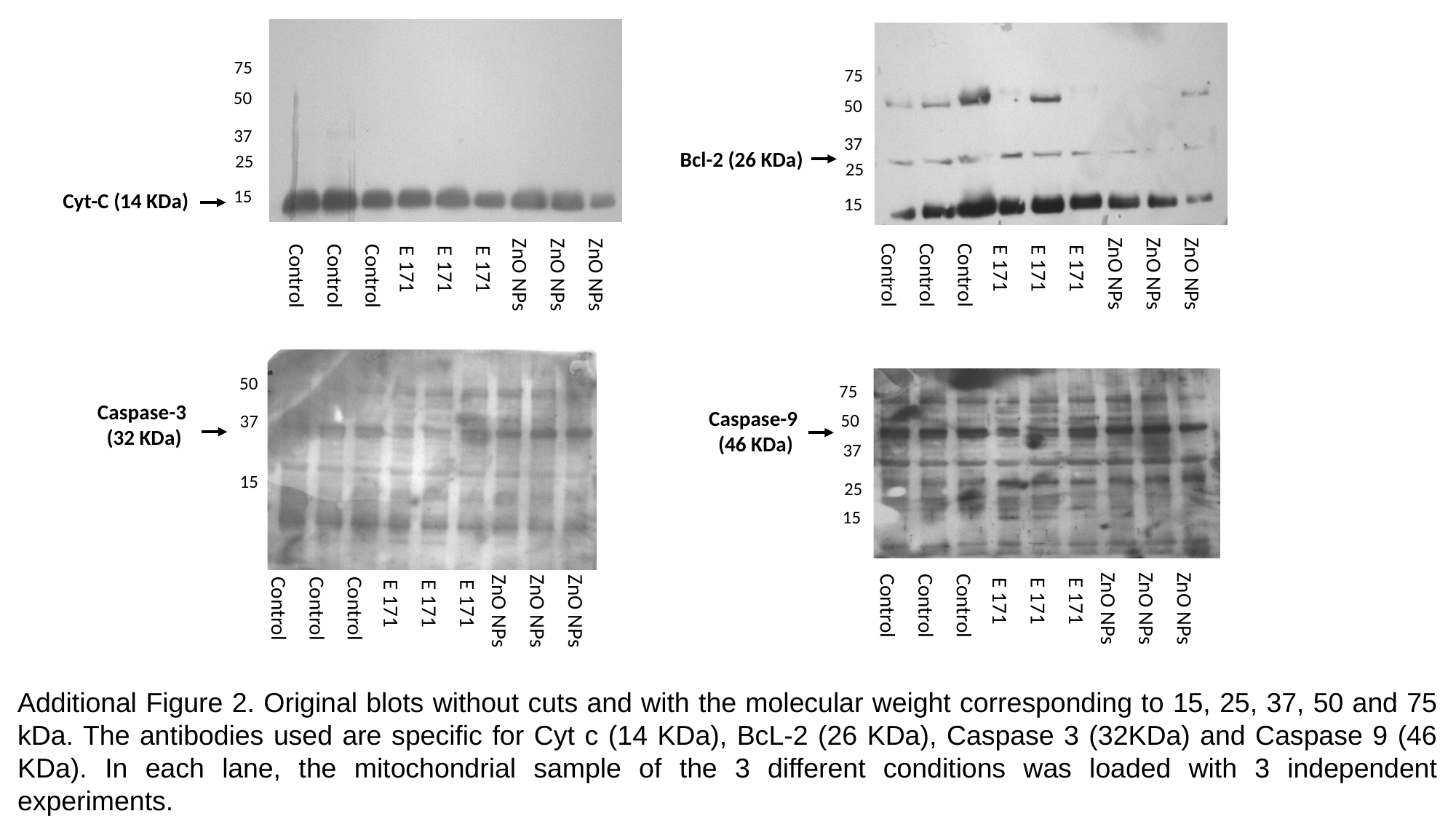

75
50
37
25
15
75
50
37
25
15
Bcl-2 (26 KDa)
Cyt-C (14 KDa)
E 171
E 171
E 171
ZnO NPs ZnO NPs ZnO NPs
Control
Control
Control
E 171
E 171
E 171
ZnO NPs
ZnO NPs
ZnO NPs
Control
Control
Control
50
37
15
75
50
37
25
15
Caspase-3
 (32 KDa)
Caspase-9
 (46 KDa)
E 171
E 171
E 171
Control
Control
Control
ZnO NPs
ZnO NPs ZnO NPs
E 171
E 171
E 171
Control
Control
Control
ZnO NPs
ZnO NPs ZnO NPs
Additional Figure 2. Original blots without cuts and with the molecular weight corresponding to 15, 25, 37, 50 and 75 kDa. The antibodies used are specific for Cyt c (14 KDa), BcL-2 (26 KDa), Caspase 3 (32KDa) and Caspase 9 (46 KDa). In each lane, the mitochondrial sample of the 3 different conditions was loaded with 3 independent experiments.
