# Supplementary material for: Food-grade titanium dioxide and zinc oxide nanoparticles induce toxicity and cardiac damage after oral exposure in rats
Source: Part Fibre Toxicol. 2023 Nov 17;20:43. doi: 10.1186/s12989-023-00553-7 (PMC10655394; doi:10.1186/s12989-023-00553-7)
Supplement: Supplementary file 3 — Additional file 3 Fig. S3. Original blots without cuts and with the molecular weight corresponding to 15, 25, 37, 50 and 75 KDa. Description of data: The antibodies used are specific for LC3B (15 KDa) and Beclin-1 (52 KDa). The Cox IV (15 KDa) and GAPDH (36 KDa) was used as a loading control. In each lane, the mitochondrial sample of the 3 different conditions was loaded with 3 independent experiments. [file 12989_2023_553_MOESM3_ESM.pptx]

## Slide 1
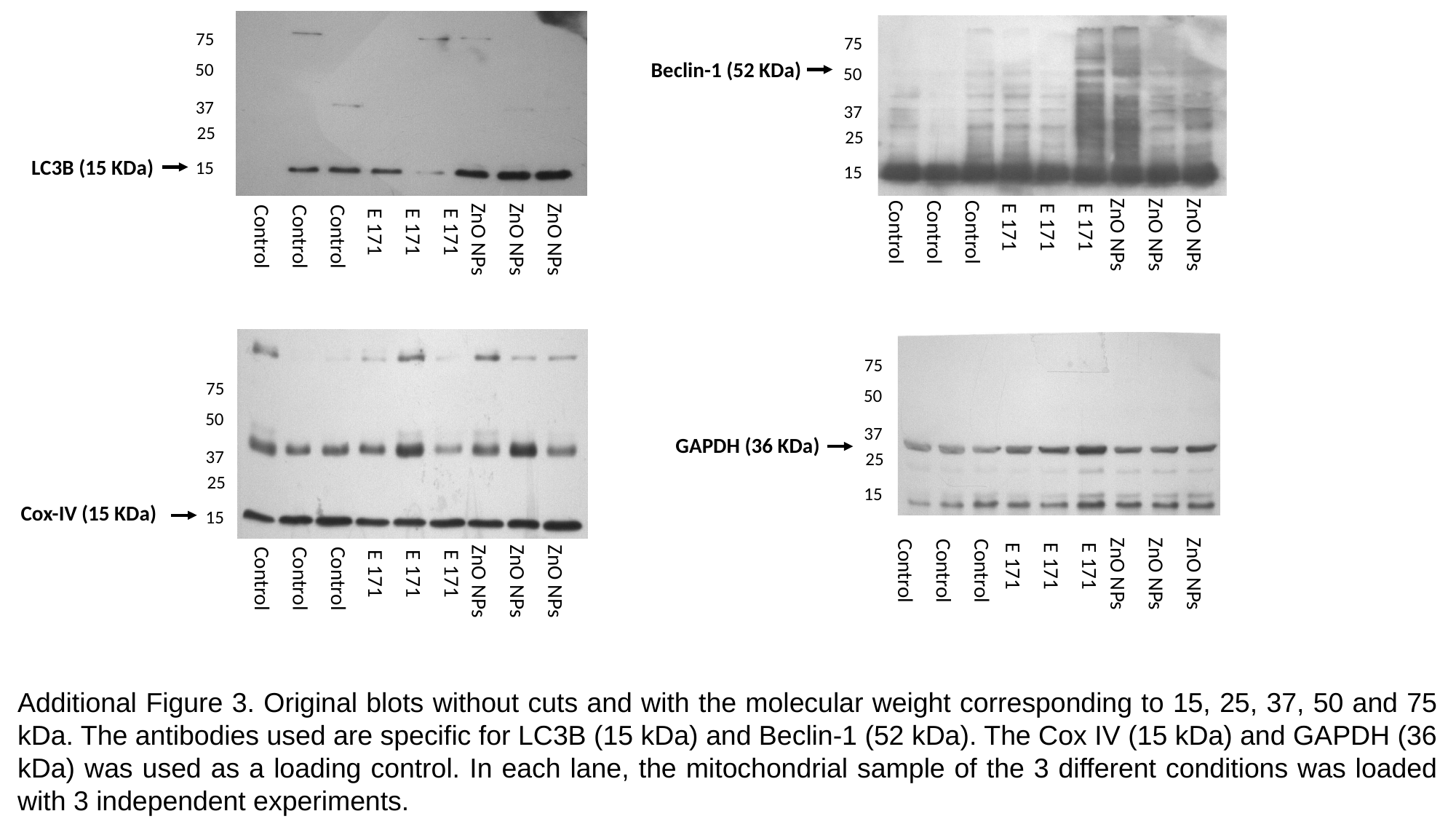

75
50
37
25
15
75
50
37
25
15
Beclin-1 (52 KDa)
LC3B (15 KDa)
E 171
E 171
E 171
Control
Control
Control
ZnO NPs ZnO NPs ZnO NPs
E 171
E 171
E 171
Control
Control
Control
ZnO NPs
ZnO NPs ZnO NPs
75
50
37
25
15
75
50
37
25
15
GAPDH (36 KDa)
Cox-IV (15 KDa)
E 171
E 171
E 171
Control
Control
Control
ZnO NPs ZnO NPs
ZnO NPs
E 171
E 171
E 171
Control
Control
Control
ZnO NPs
ZnO NPs
ZnO NPs
Additional Figure 3. Original blots without cuts and with the molecular weight corresponding to 15, 25, 37, 50 and 75 kDa. The antibodies used are specific for LC3B (15 kDa) and Beclin-1 (52 kDa). The Cox IV (15 kDa) and GAPDH (36 kDa) was used as a loading control. In each lane, the mitochondrial sample of the 3 different conditions was loaded with 3 independent experiments.
